# Vaccine Effects on Susceptibility and Symptomatology Can Change the Optimal Allocation of COVID-19 Vaccines: South Korea as an Example

**DOI:** 10.3390/jcm10132813

**Published:** 2021-06-25

**Authors:** Wongyeong Choi, Eunha Shim

**Affiliations:** Department of Mathematics, Soongsil University, 369 Sangdoro, Dongjak-Gu, Seoul 06978, Korea; chok10004@soongsil.ac.kr

**Keywords:** COVID-19, mathematical model, vaccination, optimal control theory, vaccine efficacy

## Abstract

The approved coronavirus disease (COVID-19) vaccines reduce the risk of disease by 70–95%; however, their efficacy in preventing COVID-19 is unclear. Moreover, the limited vaccine supply raises questions on how they can be used effectively. To examine the optimal allocation of COVID-19 vaccines in South Korea, we constructed an age-structured mathematical model, calibrated using country-specific demographic and epidemiological data. The optimal control problem was formulated with the aim of finding time-dependent age-specific optimal vaccination strategies to minimize costs related to COVID-19 infections and vaccination, considering a limited vaccine supply and various vaccine effects on susceptibility and symptomatology. Our results suggest that “susceptibility-reducing” vaccines should be relatively evenly distributed among all age groups, resulting in more than 40% of eligible age groups being vaccinated. In contrast, “symptom-reducing” vaccines should be administered mainly to individuals aged 20–29 and ≥60 years. Thus, our study suggests that the vaccine profile should determine the optimal vaccination strategy. Our findings highlight the importance of understanding vaccine’s effects on susceptibility and symptomatology for effective public health interventions.

## 1. Introduction

Since the first confirmed case of coronavirus disease (COVID-19) in December 2019, the disease has continued to spread worldwide and has increased not only the human health burden but also the socioeconomic burden, despite aggressive non-pharmaceutical interventions. As of 16 April 2021, 1.39 billion cases and 2.99 million deaths have been reported worldwide [1].

To mitigate local COVID-19 outbreaks in South Korea, the government promptly adopted the “test, trace, isolate, and treat” strategy and implemented non-pharmaceutical interventions, such as social distancing and mandatory mask wearing [2,3,4]. Although South Korea has been relatively successful in controlling COVID-19, ≥112,000 reported cases have been reported as of 16 April 2021 [2]. Moreover, the third wave of the COVID-19 pandemic has been ongoing in the country since mid-November 2020. This is following the first wave that mainly affected Daegu and Gyeongsangbuk-do in February–March 2020 and the second wave that mainly affected metropolitan areas in August–September 2020 [2].

COVID-19 is highly contagious and can rapidly proliferate in the absence of pre-existing immunity and non-implementation of pharmaceutical interventions; therefore, the need for vaccines to disrupt transmission and achieve herd immunity is indispensable [5]. As of 16 April 2021, 89 COVID-19 vaccines were reported in human clinical trials, including 23 in the final stages of testing [6]. Among these vaccines, the Pfizer and AstraZeneca vaccines, which require two doses, and Johnson & Johnson’s one-dose vaccine have been approved for use in South Korea [2,7]. The results of the phase III vaccine trials of Pfizer-BioNTech revealed that their vaccine could reduce the incidence of symptomatic COVID-19 by 95% [8,9,10]. The Oxford-AstraZeneca vaccine trial showed that their vaccine could reduce the incidence of symptomatic disease by approximately 70% [11]. However, the efficacy of these vaccines in preventing SARS-CoV-2 infections is still unknown. Although further research on the efficacy of COVID-19 vaccines against disease susceptibility is needed, some previous studies have suggested that infection susceptibility is reduced by more than 75% after the second dose of the Pfizer vaccine [10,12,13,14,15]. Similarly, the Johnson & Johnson vaccine trial suggested that their vaccine may reduce infection susceptibility by >70% [16]. Therefore, optimal vaccination strategies would be helpful in achieving public health goals while considering alternative scenarios for uncertain components such as the efficacy of vaccines against infection and symptom reduction.

COVID-19 vaccination strategies are dependent on public health goals. For example, the working-age population (18–59 years old) is prioritized in Indonesia since they are more likely to get infected and spread the disease [17]. In contrast, in the United States, Canada, and Israel, vaccinations are prioritized for older adults to minimize mortality and disease severity [18,19,20]. Similarly, in South Korea, health workers and the elderly were prioritized to minimize disease severity and mortality [2]. Specifically, vaccinations in South Korea began on 26 February 2021 for patients in nursing homes aged below 65 years, medical staff, caregivers, personnel of high-risk medical institutions, and medical workers at COVID-19 treatment facilities [2]. Patients ≥65 years old who were excluded from the first vaccination round due to a lack of information on the vaccine’s efficacy and side effects were vaccinated from 24 March 2021 [2]. Individuals ≤17 years old were excluded because the data on vaccine efficacy for this age group are limited. Public health authorities in South Korea aim to achieve COVID-19 herd immunity by November 2021, before the onset of the influenza epidemic [21].

As of 16 April 2021, the South Korean government has reportedly secured vaccines for 79 million people, greatly exceeding the country’s population of 52 million persons. However, even with sufficient vaccines, it is uncertain whether the vaccines will be supplied as scheduled, partially due to the high vaccine demand and production delay. The government expects that 13 million people, about 25% of the Korean population, can receive one vaccine dose in the first half of 2021 [2]. As of 16 April 2021, more than two million people have received a first AstraZeneca vaccine dose; 1.6 million people have received a first Pfizer vaccine dose; and 0.47 million people have received a second dose of either the AstraZeneca or Pfizer vaccine [2].

There are few mathematical modeling studies on optimal control strategies against COVID-19 [22,23,24,25,26,27,28,29,30]. Some of these studies focused on the dynamic prioritization of COVID-19 vaccines considering the epidemiological characteristics of COVID-19, including the impact of vaccines on infection and transmission, group heterogeneity (susceptibility, severity, and contact rates), and a wide range of plausible scenarios [23,26,27,28,29,30]. Given that demographic features, contact networks, and seroprevalence are country-specific, the optimal vaccination strategy might vary between countries. 

This study proposed an age-structured mathematical model of COVID-19 transmission with vaccination in South Korea using country-specific epidemiological data. Using optimal control theory, we identified time-dependent optimal strategies that could minimize costs associated with infection and intervention under different epidemiological scenarios such as vaccine efficacy, supply level, infectiousness of asymptomatic individuals, and vaccination capacity.

## 2. Methods

### 2.1. Epidemiological Data

Data on the cumulative number of confirmed cases up to 16 April 2021 were obtained from daily reports published by the Korea Disease Control and Prevention Agency (KDCA) (Table 1) [2]. Population distribution in age groups and age-stratified contact rates in South Korea were used to parameterize our mathematical model.

### 2.2. Mathematical Model of COVID-19 Transmission and Vaccination

**Figure 1:**
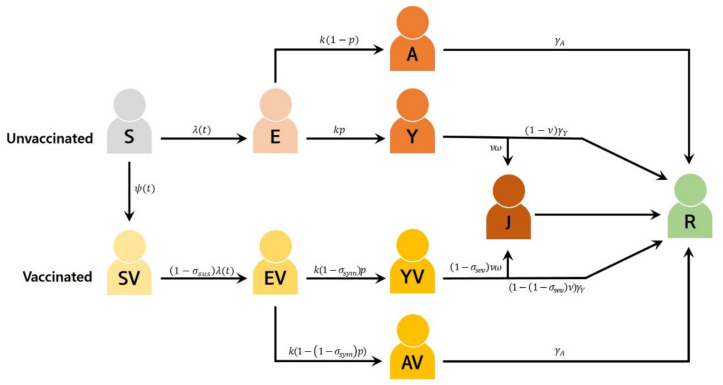
COVID-19 transmission model with vaccination. All individuals are stratified by age, although age indices have been omitted for clarity.

The age-structured mathematical model is shown in Figure 1. This model was stratified by age (0–9, 10–19, 20–29, 30–39, 40–49, 50–59, 60–69, and 70+ years), epidemiological status, and vaccination status. For each age group *i* (*i* = 1, …, 8), the model tracked susceptible ($S_{i}$), exposed ($E_{i}$), asymptomatic ($A_{i}$), symptomatic ($Y_{i}$), and severe or critical ($J_{i}$) individuals. Similarly, vaccinated individuals are divided into susceptible with partial protection ($SV_{i}$), exposed ($EV_{i}$), asymptomatic ($AV_{i}$), and symptomatic ($YV_{i}$) groups. After the infectious period, individuals are considered recovered ($R_{i}$). The $J_{i}$ groups may need treatment with high flow oxygen therapy, mechanical ventilation, extracorporeal membrane oxygenation, and continuous renal replacement therapy. The total population size is given by $N\left(t\right)=\sum_{i=1}^{8}N_{i}\left(t\right)=\sum_{i=1}^{8}\left(S_{i}\left(t\right)+SV_{i}\left(t\right)+E_{i}\left(t\right)+EV_{i}\left(t\right)+Y_{i}\left(t\right)+YV_{i}\left(t\right)+A_{i}\left(t\right)+AV_{i}\left(t\right)+J_{i}\left(t\right)+R_{i}\left(t\right)\right)$. Considering that the study period was short, natural births and deaths were ignored to ensure an asymptotically constant total population as t→∞ (i.e.,$\;N_{i}^{*}\left(t\right)=K_{i}$ and $N^{*}\left(t\right)=K$).

We assumed that susceptible individuals in age group *i* would be vaccinated at the rate of $\psi_{i}\left(t\right)$ ($0\leq \psi_{i}\left(t\right)\leq \psi_{max}$) or would progress to the exposed state at the rate of $\lambda_{i}\left(t\right)$ (Table 2). For contact patterns in South Korea, a previously published age-structured contact matrix was used [31]. The age-specific susceptibility of our model was fitted to the cumulative number of COVID-19 cases in the age groups [2].

After a latent period of 1/k on average, exposed individuals progress to infectious stages. The age-specific proportion of infections that become symptomatic is denoted by $p_{i}$ . We assumed that symptomatic individuals might be hospitalized for severe or critical disease after 1/ω days on average, with the age-specific hospitalization ratio denoted by $\nu_{i}$ . Finally, the asymptomatic and symptomatic individuals recover at the rate of $\gamma_{A}$ and $\gamma_{Y}$, respectively. Hospitalized individuals recover at the rate of $\gamma_{hosp}$ . We hypothesized that both natural and vaccine-induced immunity would last in our simulated time horizon of 400 days (i.e., T=400 ).

Based on the assumptions and definitions discussed above, our age-structured model of COVID-19 transmission and vaccination is presented as below:

$$\begin{array}{l} S_{i}^{\prime }\left(t\right)=-\lambda_{i}\left(t\right)S_{i}\left(t\right)-\psi_{i}\left(t\right)S_{i}\left(t\right),\\ E_{i}^{\prime }\left(t\right)=\lambda_{i}\left(t\right)S_{i}\left(t\right)-kE_{i}\left(t\right),\\ Y_{i}^{\prime }\left(t\right)=kp_{i}E_{i}\left(t\right)-\left(\nu_{i}\omega +\left(1-v_{i}\right)\gamma_{Y}\right)Y_{i}\left(t\right),\\ A_{i}^{\prime }\left(t\right)=k\left(1-p_{i}\right)E_{i}\left(t\right)-\gamma_{A}A_{i}\left(t\right),\\ SV_{i}{}^{\prime }\left(t\right)=\psi_{i}\left(t\right)S_{i}\left(t\right)-\left(1-\sigma_{sus}\right)\lambda_{i}\left(t\right)SV_{i}\left(t\right),\\ EV_{i}{}^{\prime }\left(t\right)=\left(1-\sigma_{sus}\right)\lambda_{i}\left(t\right)SV_{i}\left(t\right)-kEV_{i}\left(t\right),\\ YV_{i}{}^{\prime }\left(t\right)=k\left(1-\sigma_{sym}\right)p_{i}EV_{i}\left(t\right)-\left\{\left(1-\sigma_{sev}\right)\nu_{i}\omega +\left(1-\left(1-\sigma_{sev}\right)\nu_{i}\right)\gamma_{Y}\right\}YV_{i}\left(t\right),\\ AV_{i}{}^{\prime }\left(t\right)=k\left(1-\left(1-\sigma_{sym}\right)p_{i}\right)EV_{i}\left(t\right)-\gamma_{A}AV_{i}\left(t\right),\\ J\prime_{i}\left(t\right)=\nu_{i}\omega \left(Y_{i}\left(t\right)+\left(1-\sigma_{sev}\right)YV_{i}\left(t\right)\right)-\gamma_{hosp}J_{i}\left(t\right),\\ R_{i}^{\prime }\left(t\right)=\gamma_{Y}\left\{\left(1-v_{i}\right)Y_{i}\left(t\right)+\left(1-\left(1-\sigma_{sev}\right)\nu_{i}\right)YV_{i}\left(t\right)\right\}+\gamma_{A}\left(AV_{i}\left(t\right)+A_{i}\left(t\right)\right)+\gamma_{hosp}J_{i}\left(t\right), \end{array}$$

where the force of infection for a susceptible individual in the age group i, $\lambda_{i}\left(t\right)$ , is given by

$$\lambda_{i}\left(t\right)=\beta_{0}u_{i}\overset{8}{\underset{j=1}{\sum }}c_{ij}\frac{Y_{j}\left(t\right)+YV_{j}\left(t\right)+b\left(A_{j}\left(t\right)+AV_{j}\left(t\right)\right)}{N_{j}\left(t\right)}.$$

where $c_{ij}$ is the number of age- j individuals contacted by an age- i individual per day, $u_{i}$ is the age-specific susceptibility, and b is the relative infectiousness of asymptomatic individuals compared with symptomatic individuals.

### 2.3. Basic Reproduction Number

The basic reproduction number $\left(R_{0}\right)$ represents the average number of secondary infections among the entire susceptible population caused by one infected individual, in the absence of control measures (i.e., $\psi_{i}\left(t\right)=0$). We defined the next-generation matrix $FW^{-1}$ as

$$FW^{-1}={\left(M_{ij}\right)}_{i,j=1,\ldots ,8}$$

where

$$M_{ij}=\beta_{0}u_{i}c_{ij}\left(\frac{b\left(1-p_{j}\right)}{\gamma_{A}}+\frac{p_{j}}{\nu_{j}\omega +\left(1-v_{j}\right)\gamma_{Y}}\right)\frac{S_{i}\left(0\right)}{N_{j}}.$$

Then, it follows that $R_{0}=\rho \left(FW^{-1}\right)$, where ρ denotes spectral radius. Our study focused on scenarios with a partially mitigated pandemic ($R_{0}=1.3$), consistent with control reproductive ratio estimates in South Korea. The baseline values for epidemiological parameters (Table 2) were used for the simulations, unless otherwise specified.

### 2.4. Vaccination Scenarios

We assumed that the use of a leaky vaccine may have three probable effects on vaccinated individuals: reducing the infection rate among vaccinated individuals by $\sigma_{sus}$, decreasing the probability of progression to symptomatic disease by $\;\sigma_{sym}$, and reducing the likelihood of progression to severe or critical disease by $\sigma_{sev}$. Thus, the multiplicative reduction in the risk of disease per exposure, denoted by σ, was calculated as $\sigma =1-\left(1-\sigma_{sym}\right)\left(1-\sigma_{sus}\right)$ [46]. Subsequently, vaccinated individuals might be infected at the rate of $\left(1-\sigma_{sus}\right)\lambda_{i}\left(t\right)$, and upon infection, the fraction $\left(1-\sigma_{sym}\right)p_{i}$ of individuals would experience symptoms.

Given the efficacy data of two-dose COVID-19 vaccines such as Oxford-AstraZeneca, which has been most widely used in South Korea to date, we considered a main scenario with a 70% disease risk reduction [11]. Since many combinations of $\sigma_{sus}$ and $\sigma_{sym}$ can result in the same σ, we considered three vaccine profiles that yield σ=0.7: a vaccine effect mediated by susceptibility reduction ($\sigma_{sus}$) only (vaccine 1), symptomatology reduction ($\sigma_{sym}$) only (vaccine 3), and a combination of $\sigma_{sus}$ and $\sigma_{sym}$ (vaccine 2) (Table 3).

A 95% disease risk reduction was considered in the sensitivity analysis, consistent with the effectiveness of Novavax [43], Pfizer-BioNTech [8], and Moderna [9] vaccines. Similarly, three vaccine profiles were considered: a vaccine effect mediated by susceptibility reduction ($\sigma_{sus}$) only (vaccine 4), symptomatology reduction ($\sigma_{sym}$) only (vaccine 6), and a combination of $\sigma_{sus}$ and $\sigma_{sym}$ (vaccine 5) (Table 3). For all the vaccine profiles, we assumed that $\sigma_{sev}=0.9$ [9,10,12,43].

We considered that 50% of the total population would receive two doses at baseline. The optimal vaccination strategy for a higher vaccination coverage (70% of South Korean population, or equivalently, 85% of the vaccine-eligible population) was assessed in the sensitivity analysis. All vaccines were assumed to take effect immediately after their administration.

### 2.5. Formulation of the Optimal Control Problem

This study aimed to minimize costs of COVID-19 infection and vaccination over 400 days (*T* = 400). Considering a limited vaccine supply, the model was developed in the framework of a constrained optimal control problem. Specifically, the objective functional, F , to be minimized was

$$F\left(\psi_{i}\left(t\right)\right)=\int_{t=0}^{T}\overset{8}{\underset{i=1}{\sum }}\left\{C_{Vac}\psi_{i}^{2}\left(t\right)s_{i}\left(t\right)+C_{Y}\left(y_{i}\left(t\right)+yv_{i}\left(t\right)\right)+C_{hosp}j_{i}\left(t\right)\right\}dt$$

where the control effort is modelled by quadratic terms in $\psi_{i}\left(t\right).$ To reflect the fact that children and adolescents are ineligible for vaccination, we assumed that people <20 years would not be vaccinated ($\psi_{1}\left(t\right)=\psi_{2}\left(t\right)=0$). Here, $C_{Vac}$ denotes the cost of vaccination, while $C_{Y}$ and $C_{hosp}$ are the daily costs associated with mildly symptomatic infectious individuals and hospitalized individuals, respectively. The constrained optimal problem with the isoperimetric constraint (i.e., a limited vaccine supply) consisted of finding the age-dependent optimal vaccination strategies for COVID-19, $\psi^{*}\left(t\right)$, such that

$$F\left(\psi_{i}^{*}\left(t\right)\right)=\underset{\Theta }{\min}F\left(\psi_{i}\left(t\right)\right)$$

where $\int_{0}^{T}\overset{8}{\underset{i=1}{\sum }}\psi_{i}\left(t\right)s_{i}\left(t\right)dt=B,$ $\Theta =\left\{\psi_{i}\left(t\right)\in L^{1}\left(0,T\right)|0\leq \psi_{i}\leq \psi_{max}\right\}$, and subject to our model. We assumed that each age-specific vaccination rate, $\psi_{i}\left(t\right)$, is bound by the maximum rate, $\psi_{max}$. To solve this problem numerically, we introduced an extra variable $z\left(t\right)$ with $z^{\prime }\left(t\right)=\overset{8}{\underset{i=3}{\sum }}\psi_{i}\left(t\right)s_{i}\left(t\right)$, $z\left(0\right)=0,$ and $z\left(T\right)=B$, which expresses the cumulative proportion of vaccinated individuals, allowing us to include the isoperimetric constraint and to apply Pontryagin’s maximum principle to our model [47,48] (Appendix A).

The Hamiltonian H is minimized with respect to the controls at the optimal rates, giving the following optimality conditions:

$${\left.\frac{\partial H}{\partial \psi_{i}}\right|}_{\psi_{i}\left(t\right)=\psi_{i}^{*}}=0.$$

By solving for $\psi_{i}$ , we obtain

$$\psi_{i}^{*}=\min\left\{max\left\{0,\;\frac{\xi_{s_{i}}-\xi_{sv_{i}}-\xi_{z}}{2C_{Vac}}\right\},\psi_{max}\;\right\}.$$

Numerical simulations of the optimal COVID-19 vaccination strategies based on the proposed mathematical model were performed.

## 3. Results

### 3.1. Optimal Immunization Strategy with Vaccines That Reduces the Risk of COVID-19 by 70%

**Figure 2:**
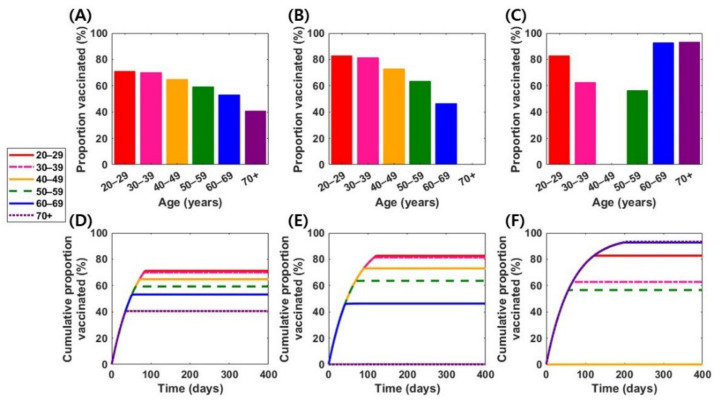
Optimal vaccination strategies in various vaccine scenarios. The first row represents age-specific levels of optimal vaccination coverage in the vaccine 1 (**A**), vaccine 2 (**B**), and vaccine 3 (**C**) scenarios. The second row represents time-dependent cumulative vaccination coverage levels under optimal vaccination schemes in the vaccine 1 (**D**), vaccine 2 (**E**), and vaccine 3 (**F**) scenarios.

With the baseline parameter values and in the absence of vaccination, the cumulative proportion of infected individuals reached 43%. The optimal control problem of limited vaccine supply in South Korea was solved with vaccine profiles 1, 2, and 3 ( Table 3). A “susceptibility-reducing” vaccine with $\sigma_{sus}=0.7$ and no effects on reducing symptoms ($\sigma_{sym}=0$) can prevent 98% of symptomatic infections and hospitalization over 400 days, where more than 40% of individuals in every vaccine-eligible age group would be vaccinated under optimal vaccination schemes (Figure S1). Considering this scenario, 71% of adults aged 20–29, 70% of adults aged 30–39, and 65% of adults aged 40–49 would be vaccinated (Figure 2A).

In comparison, persons aged 20–29 and ≥60 years were vaccinated with a “symptom-reducing” vaccine (i.e., vaccine profile 3 in Table 3) for direct protection, resulting in vaccination coverage levels >82% in those age groups (Figure 2C). In this case, a vaccine that can only prevent symptoms can prevent hospitalizations and symptomatic infections by only 82% and 54%, respectively (Figure S1).

Finally, if both symptoms and infection prevention were attained, prioritizing persons aged 20-39 while vaccinating 46% of 60–69-year-old individuals would be an optimal strategy (Figure 2B). Compared with the scenario of the “symptom-reducing” vaccine, adults aged 30–59 years are more likely to be vaccinated, and those aged ≥60 years are less likely to be vaccinated.

### 3.2. Impact of a Higher Vaccine Efficacy

**Figure 3:**
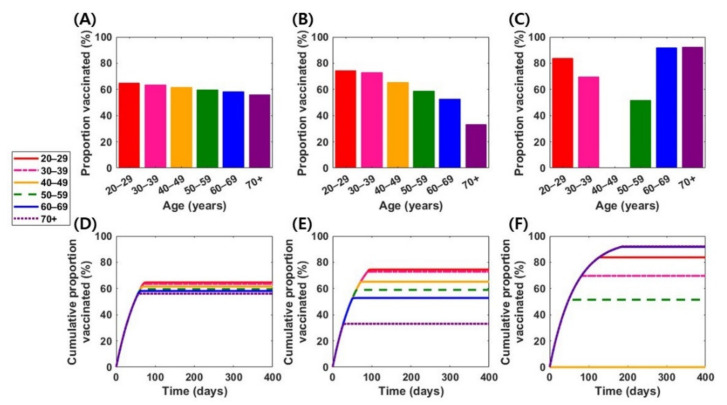
Optimal vaccination strategies in various vaccine scenarios when vaccines provide 95% of reduction in the risk of disease per exposure. The first row represents age-specific levels of optimal vaccination coverage in the vaccine 4 (**A**), vaccine 5 (**B**), and vaccine 6 (**C**) scenarios. The second row represents time-dependent cumulative vaccination coverage levels under optimal vaccination schemes in the vaccine 4 (**D**), vaccine 5 (**E**), and vaccine 6 (**F**) scenarios.

The effect of a vaccine with a higher efficacy, σ=0.95, was assessed in sensitivity analysis, considering the efficacy of two mRNA-based vaccines. In this section, three vaccine profiles (vaccines 4, 5, and 6; Table 3) were considered: “susceptibility-reducing” vaccine ($\sigma_{sus}=0.95$ and $\sigma_{sym}=0$), “symptom-reducing” vaccine ($\sigma_{sus}=0$ and $\sigma_{sym}=0.95$), and a vaccine with moderate effects on the reduction in both susceptibility and symptoms ($\sigma_{sus}=0.50$ and $\sigma_{sym}=0.90$). Using a “susceptibility-reducing” vaccine, vaccine doses were relatively evenly allocated to all vaccine-eligible individuals, reducing more than 99% of the symptomatic infections and hospitalizations (Figure S2). However, more vaccine doses should be allocated to individuals aged 20–29 and ≥60 years if a symptom-reducing vaccine is used, while the remaining doses should be administered to persons aged 30–39 and 50–59 years (Figure 3C). The optimal vaccination strategy with a vaccine having moderate effects is the relatively even allocation of vaccines among age groups, but with higher coverage levels for individuals aged 20–39 years (Figure 3B).

### 3.3. Impact of Higher Vaccine Supply Level

**Figure 4:**
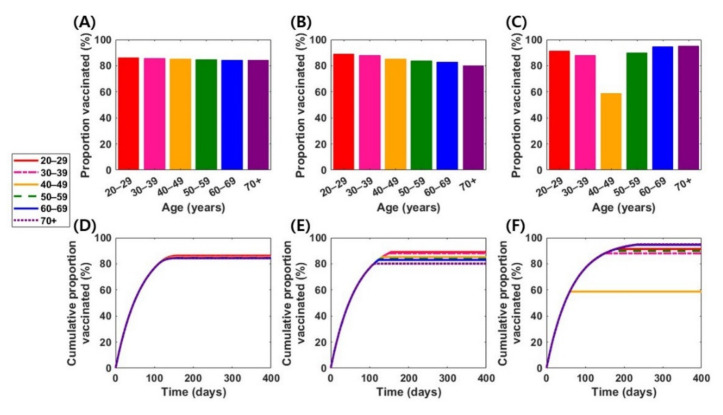
Optimal vaccination strategies in various vaccine scenarios with higher vaccine supply level (B = 70%). The first row represents age-specific levels of optimal vaccination coverage in the vaccine 1 (**A**), vaccine 2 (**B**), and vaccine 3 (**C**) scenarios. The second row represents time-dependent cumulative vaccination coverage levels under optimal vaccination schemes in the vaccine 1 (**D**), vaccine 2 (**E**), and vaccine 3 (**F**) scenarios.

Assuming that vaccines are available to immunize 70% of the South Korean population (or 85% of the vaccine-eligible population), the main analysis was repeated. Using a “susceptibility-reducing” vaccine and a vaccine with moderate effects, optimal vaccination coverage levels were approximately similar among all age groups, leading to a coverage level >80% of all vaccine-eligible individuals regardless of age group (Figure 4). Additionally, “symptom-reducing” vaccines would be distributed to >58% of adults aged 40–49 years who may not be vaccinated under the limited vaccine supply scenario, and >88% of other age groups (Figure 4C).

### 3.4. Impact of Relative Infectiousness of Asymptomatic Infections

**Figure 5:**
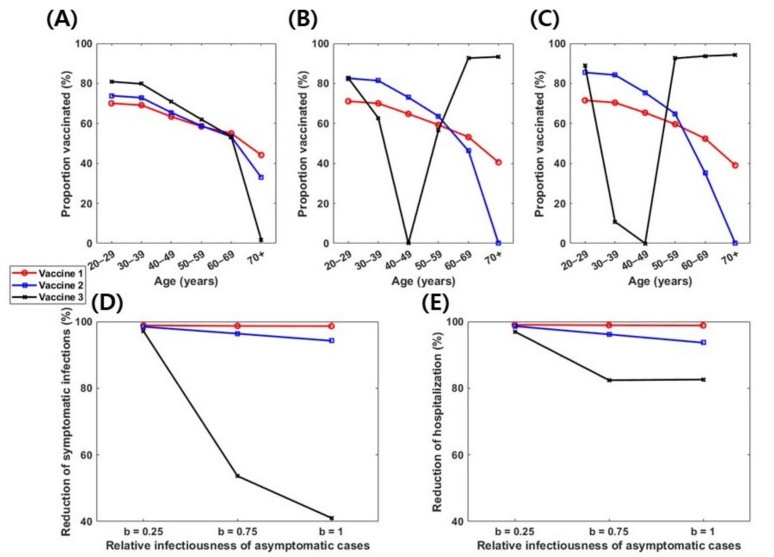
Effect of the relative infectiousness of asymptomatic infections on optimal vaccination strategies. (**A**–**C**) Age-specific optimal vaccination coverage levels with vaccines 1, 2, and 3, when b = 0.25 (**A**), 0.75 (**B**), and 1 (**C**). (**D**) Corresponding proportion of reduction in number of patients with symptomatic infections relative to non-vaccinated cases. (**E**) Corresponding proportion of reduction in hospitalizations relative to non-vaccination cases.

Given the wide range of estimates of the relative infectiousness of asymptomatic infections compared to symptomatic ones (0.2–1) [49], the analysis was repeated with an estimated relative infectiousness of asymptomatic infections at 25% and 100%, i.e., *b*=0.25 and 1, respectively (Figure 5). Overall, with a lower transmissibility of asymptomatic infection (*b*=0.25), the differences in optimal vaccination coverage levels among age groups would be lower, preventing up to 98% of symptomatic infections and hospitalizations, regardless of vaccine efficacy profiles (Figure 5). Conversely, if there was no difference in infectiousness between symptomatic and asymptomatic infections (b=1), vaccine 3 would be optimal for younger adults aged 20–29 years and adults >50 years old (Figure 5C,F).

### 3.5. Impact of Daily Vaccination Capacity

**Figure 6:**
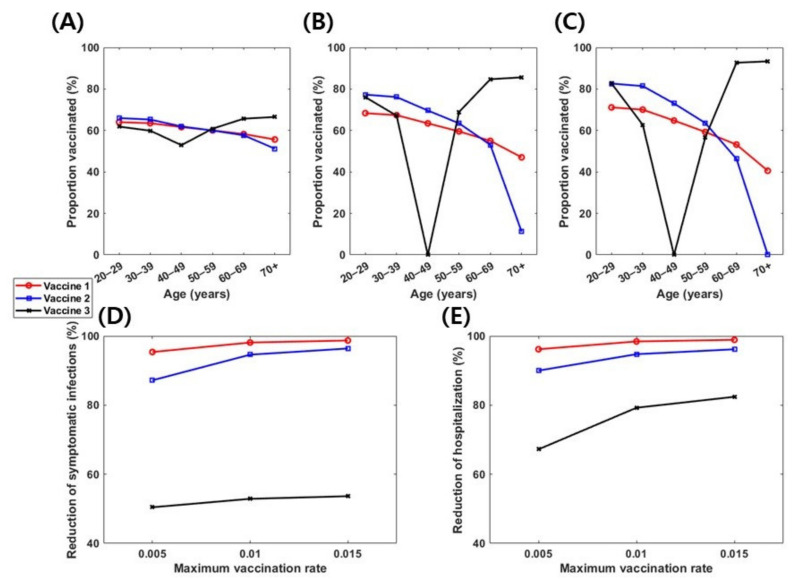
Effect of vaccine rollouts on optimal vaccination strategies. (**A**–**C**) Age-specific optimal vaccination coverage levels in various vaccine efficacy profiles (1, 2, and 3) with $\psi_{max}=$ 0.005 (**A**), 0.01 (**B**), and 0.015 (**C**). (**D**) Corresponding proportion of reduction in symptomatic infections relative to cases without vaccination. (**E**) Corresponding proportion of reduction in hospitalization relative to cases without vaccination.

We also investigated the importance of daily vaccination capacity by computing the optimal vaccination strategies when the upper bound of daily vaccination rates ($\psi_{max})$ was 0.005 and 0.01 (Figure 6). With a lower daily vaccination capacity, optimal vaccine allocation would more likely be evenly distributed among age groups. For instance, when $\psi_{max}=0.005$, the optimal vaccination coverage levels for all vaccine-eligible individuals were >51% regardless of vaccine profiles (Figure 6). The reduction in the number of symptomatic infections and hospitalizations decreased when $\psi_{max}$ decreased, especially with vaccine 3 (Figure 6D,E).

## 4. Discussion

This study explored different combinations of vaccine effects and derived optimal COVID-19 vaccination strategies in South Korea. These strategies may be helpful in minimizing vaccination and infection costs, including the cost associated with symptomatic infections and treating people with severe disease. Various vaccine effects with a 70% reduction in symptomatic COVID-19 infections and a limited vaccine supply were considered. The study results indicate that the vaccination rates for all age groups should be at their maximum in the early stages, which is consistent with previous studies [47,50,51,52]. Overall, the optimal vaccination strategy greatly reduced the total number of hospitalized cases (≥80%).

There should be a relatively even distribution of “susceptibility-reducing” vaccines among all age groups, while “symptom-reducing” vaccines should be reserved for individuals aged 20–29 and 60+ years. Age-specific optimal policies were relatively insensitive to vaccine efficacy (*σ* = 70% or 95%) or the vaccine supply level (*B* = 50% or 70%); however, the most noticeable differences were found when the effects of different vaccine profiles were analyzed.

There are several published studies on optimal age-specific vaccination prioritization strategies with varying vaccine supply levels and efficacies [26,28,29,30,53,54]. Some studies found that prioritizing “susceptibility-reducing” vaccines for young adults minimized the cumulative incidence, and minimized mortality and years of life lost when prioritized for older adults [31,32,39,54]. Considering all impacts of the vaccine, most younger adults (aged 20–29 years) should always be vaccinated, since younger adults are active, and thus responsible for a greater part of disease transmission. This is consistent with prior findings by Matrajt et al. that concluded that allocating vaccines to younger age groups would be optimal in minimizing infections and hospitalizations, if vaccine efficacy is relatively high [26]. If the vaccine reduces symptoms only, it is optimal to vaccinate a greater number of older adults (≥60 years), since they are more likely to experience serious symptoms than other age groups. Similarly, a prior study based on data from the United Kingdom suggested that targeting older age groups first is optimal with the aim of minimizing future deaths or quality-adjusted life year losses, if the vaccine prevents transmission as well as disease [23]. In addition, Cartocci et al. found that, in Italy, vaccinating the older age groups would be optimal in reducing total deaths [55].

This study has several limitations. First, we assumed that vaccine-eligible individuals would actively participate in vaccination so that early-phase vaccine rollout rates can be maximized to meet the daily requirements. However, it might be difficult to administer vaccines in a large scale especially in early phases due to a lack of medical facilities and human resources or vaccine hesitancy. Second, our model assumed that vaccine-induced immunity does not wane during the simulated time horizon. Although Moderna and Pfizer-BioNTech vaccines are reported to have an efficacy above 90%, vaccine-induced immunity might wane over time, potentially changing the optimal vaccination strategy. Third, the population was stratified only by age. However, the optimal vaccination strategy may be different for healthcare workers who are frequently in contact with people having comorbidities or with older adults. Some studies which prioritized vaccinations for healthcare workers suggest that they should be targeted first [28,35]. Furthermore, some countries including the United States, Canada, Italy, France, Germany, and Japan approved vaccinating adolescents against COVID-19, making it necessary to modify the modeling assumptions and age groups accordingly. Finally, we assumed that the level of non-pharmaceutical interventions (such as social distancing and mask wearing) was steady within the simulated time horizon. However, anti-vaccination sentiment may result in a decrease in vaccine coverage and physical interactions may increase due to policy changes or reduced alertness to infections, making it necessary to readjust the optimal vaccination strategy with the increase in infection spread.

In conclusion, different vaccine profiles would significantly affect the prioritization of vaccines. Symptom-reducing vaccines should be prioritized among persons aged 20–29 and ≥60 years; and susceptibility-reducing vaccines should be relatively evenly distributed among all age groups. Although we have not considered other population structures or social aspects (returning to school, non-mandatory face mask wearing, etc.), this study adds to the existing knowledge on optimal vaccination strategies.

## Figures and Tables

**Table 1:** Cumulative number of cases grouped by age in South Korea (as of 16 April 2021).

		Confirmed Cases
Total		112,789
Age Group	≥70	13,257
	60–69	17,431
	50–59	20,887
	40–49	16,586
	30–39	15,161
	20–29	16,817
	10–19	7813
	0–9	4837

**Table 2:** Baseline parameter values and description. (* 1 USD = 1114 WON, as of April 16, 2021).

Parameter	Description	Value	References
$R_{0}$	Basic reproduction number	1.3	
$\beta_{0}$	Transmission probability	-	Estimated from $R_{0}$
$u_{i}$	Age-specific susceptibility	[0.26; 0.15; 0.3; 0.24; 0.22; 0.29; 0.66; 0.8]	Fitted
$c_{ij}$	Number of age- j individuals contacted by an age- i individual per day	Table S1	[31]
$p_{i}$	Age-specific proportion of symptomatic infection	[0.66; 0.62; 0.67; 0.67; 0.67; 0.67; 0.75; 0.81]	[32,33,34,35]
1/k	Latent period (day)	3.1	[36]
b	Relative infectiousness of asymptomatic individuals compared with symptomatic individuals	0.75	[26,30]
$\nu_{i}$	Age-specific hospitalization ratio of symptomatic individuals (%)	[0.23; 0.33; 0.76; 0.76; 0.76; 3.13; 5.64; 8.14]	[35,37,38,39,40]
1/ω	Average duration from diagnosis to hospitalization because of severe symptoms (day)	7	[2]
$1/\gamma_{A}$	Infectious period of asymptomatic infections (day)	7	[41,42]
$1/\gamma_{Y}$	Infectious period of symptomatic infections (day)	7	[41,42]
$1/\gamma_{hosp}$	Time spent in hospital (day)	22	[2]
$\sigma_{sev}$	Vaccine efficacy in reducing the probability that the infection progresses to severe or critical disease	0.90	[9,10,12,43]
$\psi_{max}$	Upper bound of daily vaccination rate	0.015	Assumption
$C_{V}$	Cost of vaccination for COVID-19 (USD *)	35	[2]
$C_{Y}$	Daily cost of treatment for mild infections (USD *)	197	[44]
$C_{hosp}$	Daily cost of treatment during hospitalization (USD *)	583	[44]
$n_{i}$	Proportion of people in age group i ( $n_{i}=N_{i}/K)$	$n_{1}$ = 0.08, $n_{2}$ = 0.10, $n_{3}$ = 0.13, $n_{4}$ = 0.14, $n_{5}$ = 0.16, $n_{6}$ = 0.17, $n_{7}$ = 0.12, $n_{8}$ = 0.10.	[45]

**Table 3:** Vaccine efficacy scenarios used in the main analysis.

Vaccine Efficacy Scenarios	Vaccine 1	Vaccine 2	Vaccine 3	Vaccine 4	Vaccine 5	Vaccine 6
Reduction in susceptibility ($\sigma_{sus}$)	0.70	0.40	0.00	0.95	0.50	0.00
Reduction in the probability of disease progression to symptomatic disease ($\sigma_{sym}$)	0.00	0.50	0.70	0.00	0.90	0.95
Direct impact of reduction in the number of symptomatic infections ($\sigma =1-\left(1-\sigma_{sus}\right)\left(1-\sigma_{sym}\right)$)	0.70			0.95		

## Data Availability

Publicly available datasets were analyzed in this study. This data can be found here: https://www.cdc.go.kr/board/board.es?mid=a30402000000&bid=0030 (accessed on 16 April 2021).

## Supplementary Materials

The following are available online at https://www.mdpi.com/article/10.3390/jcm10132813/s1, Table S1: Contact matrix, Figure S1: Optimal vaccination strategies in various vaccine scenarios. (A–C) Calculated optimal vaccination rates with vaccines 1 (A), 2 (B), and 3 (C). (D–F) Corresponding daily hospitalizations without immunization (dashed line) and with immunization (solid line) using vaccines 1 (D), 2 (E), and 3 (F). Figure S2: Optimal vaccination strategies in various vaccine scenarios. (A–C) Calculated optimal vaccination rates with vaccines 4 (A), 5 (B), and 6 (C). (D–F) Corresponding daily hospitalizations without immunization (dashed line) and with immunization (solid line) using vaccines 4 (D), 5 (E), and 6 (F). Figure S3: Optimal vaccination strategies in various vaccine scenarios with higher vaccine supply level (B=70%). (A–C) Calculated optimal vaccination rates with vaccines 1 (A), 2 (B), and 3 (C). (D–F) Corresponding daily hospitalizations without immunization (dashed line) and with immunization (solid line) using vaccines 1 (D), 2 (E), and 3 (F).

## Appendix A

Application of Optimal Control Theory

From our mathematical model of COVID-19 transmission and vaccination, we note that the variable $R_{i}\left(t\right)$ appears only in $R_{i}^{\prime }\left(t\right)$ , and thus we determine it after solving for the other classes. Additionally, we have a normalized model and changed the variables to

$$\begin{array}{l} s_{i}\left(t\right)=\frac{S_{i}\left(t\right)}{K},\;sv_{i}\left(t\right)=\frac{SV_{i}\left(t\right)}{K},e_{i}\left(t\right)=\frac{E_{i}\left(t\right)}{K},\;ev_{i}\left(t\right)=\frac{EV_{i}\left(t\right)}{K},\\ y_{i}\left(t\right)=\frac{Y_{i}\left(t\right)}{K},\;yv_{i}\left(t\right)=\frac{YV_{i}\left(t\right)}{K},a_{i}\left(t\right)=\frac{A_{i}\left(t\right)}{K},\;av_{i}\left(t\right)=\frac{AV_{i}\left(t\right)}{K},j_{i}\left(t\right)=\frac{J_{i}\left(t\right)}{K}, \end{array}$$

which leads us to the following system:

$$\begin{array}{l} s_{i}^{\prime }\left(t\right)=-\lambda_{i}\left(t\right)s_{i}\left(t\right)-\psi_{i}\left(t\right)s_{i}\left(t\right),\\ e_{i}^{\prime }\left(t\right)=\lambda_{i}\left(t\right)s_{i}\left(t\right)-ke_{i}\left(t\right),\\ y_{i}^{\prime }\left(t\right)=kp_{i}e_{i}\left(t\right)-\left(\nu_{i}\omega +\left(1-\nu_{i}\right)\gamma_{Y}\right)y_{i}\left(t\right),\\ a_{i}^{\prime }\left(t\right)=k\left(1-p_{i}\right)e_{i}\left(t\right)-\gamma_{A}a_{i}\left(t\right),\\ sv_{i}{}^{\prime }\left(t\right)=\psi_{i}\left(t\right)s_{i}\left(t\right)-\left(1-\sigma_{sus}\right)\lambda_{i}\left(t\right)sv_{i}\left(t\right),\\ ev_{i}{}^{\prime }\left(t\right)=\left(1-\sigma_{sus}\right)\lambda_{i}\left(t\right)sv_{i}\left(t\right)-kev_{i}\left(t\right),\\ yv_{i}{}^{\prime }\left(t\right)=kp_{i}\left(1-\sigma_{sym}\right)ev_{i}\left(t\right)-\left\{\left(1-\sigma_{sev}\right)\nu_{i}\omega +\left(1-\left(1-\sigma_{sev}\right)\nu_{i}\right)\gamma_{Y}\right\}yv_{i}\left(t\right),\\ av_{i}{}^{\prime }\left(t\right)=k\left(1-\left(1-\sigma_{sym}\right)p_{i}\right)ev_{i}\left(t\right)-\gamma_{A}av_{i}\left(t\right),\\ j_{i}\prime \left(t\right)=\nu_{i}\omega \left(y_{i}\left(t\right)+\left(1-\sigma_{sev}\right)yv_{i}\left(t\right)\right)-\gamma_{hosp}j_{i}\left(t\right), \end{array}$$

where $\lambda_{i}\left(t\right)=\beta_{0}u_{i}\sum_{j=1}^{8}c_{ij}\frac{y_{j}\left(t\right)+yv_{j}\left(t\right)+b\left(a_{j}\left(t\right)+av_{j}\left(t\right)\right)}{n_{j}}.$

From the model above, the corresponding Hamiltonian function is defined as

$$\begin{array}{l} H=\overset{8}{\underset{i=1}{\sum }}\left\{C_{Vac}\psi_{i}^{2}\left(t\right)s_{i}\left(t\right)+C_{Y}\left(y_{i}\left(t\right)+yv_{i}\left(t\right)\right)+C_{hosp}j_{i}\left(t\right)\right\}\\ +\overset{8}{\underset{i=1}{\sum }}\xi_{s_{i}}\left(t\right)\left\{-\lambda_{i}\left(t\right)s_{i}\left(t\right)-\psi_{i}\left(t\right)s_{i}\left(t\right)\right\}\\ +\overset{8}{\underset{i=1}{\sum }}\xi_{e_{i}}\left(t\right)\left\{\lambda_{i}\left(t\right)s_{i}\left(t\right)-ke_{i}\left(t\right)\right\}\\ +\overset{8}{\underset{i=1}{\sum }}\xi_{y_{i}}\left(t\right)\left\{kp_{i}e_{i}\left(t\right)-\left(\nu_{i}\omega +\left(1-\nu_{i}\right)\gamma_{Y}\right)y_{i}\left(t\right))\right\}\\ +\overset{8}{\underset{i=1}{\sum }}\xi_{a_{i}}\left(t\right)\left\{k\left(1-p_{i}\right)e_{i}\left(t\right)-\gamma_{A}a_{i}\left(t\right)\right\}\\ +\overset{8}{\underset{i=1}{\sum }}\xi_{sv_{i}}\left(t\right)\left\{\psi_{i}\left(t\right)s_{i}\left(t\right)-\left(1-\sigma_{sus}\right)\lambda_{i}\left(t\right)sv_{i}\left(t\right)\right\}\\ +\overset{8}{\underset{i=1}{\sum }}\xi_{ev_{i}}\left(t\right)\left\{\left(1-\sigma_{sus}\right)\lambda_{i}\left(t\right)sv_{i}\left(t\right)-kev_{i}\left(t\right)\right\}\\ +\overset{8}{\underset{i=1}{\sum }}\xi_{yv_{i}}\left(t\right)\left\{k\left(1-\sigma_{sym}\right)p_{i}ev_{i}\left(t\right)-\left\{\left(1-\sigma_{sev}\right)\nu_{i}\omega +\left(1-\left(1-\sigma_{sev}\right)\nu_{i}\right)\gamma_{Y}\right\}yv_{i}\left(t\right)\right\}\\ +\overset{8}{\underset{i=1}{\sum }}\xi_{av_{i}}\left(t\right)\left\{k\left(1-\left(1-\sigma_{sym}\right)p_{i}\right)ev_{i}\left(t\right)-\gamma_{A}av_{i}\left(t\right)\right\}\\ +\overset{8}{\underset{i=1}{\sum }}\xi_{j_{i}}\left(t\right)\left\{\nu_{i}\omega \left(y_{i}\left(t\right)+\left(1-\sigma_{sev}\right)yv_{i}\left(t\right)\right)-\gamma_{hosp}j_{i}\left(t\right)\right\}\\ +\xi_{z}\left(t\right)\left\{\overset{8}{\underset{i=1}{\sum }}\psi_{i}\left(t\right)s_{i}\left(t\right)\right\}. \end{array}$$

From this Hamiltonian and Pontryagin’s maximum principle, we obtain the following adjoint system:

$$\begin{array}{l} \frac{d\xi_{s_{i}}}{dt}=-\frac{\partial H}{\partial s_{i}}=-C_{Vac}\psi_{i}^{2}\left(t\right)+\left(\xi_{s_{i}}\left(t\right)-\xi_{sv_{i}}\left(t\right)-\xi_{z}\left(t\right)\right)\psi_{i}\left(t\right)+\left(\xi_{s_{i}}\left(t\right)-\xi_{e_{i}}\left(t\right)\right)\lambda_{i}\left(t\right)\\ \frac{d\xi_{sv_{i}}}{dt}=-\frac{\partial H}{\partial sv_{i}}=\left(1-\sigma_{sus}\right)\lambda_{i}\left(t\right)\left(\xi_{sv_{i}}\left(t\right)-\xi_{ev_{i}}\left(t\right)\right)\\ \frac{d\xi_{e_{i}}}{dt}=-\frac{\partial H}{\partial e_{i}}=k\left(\xi_{e_{i}}\left(t\right)-p_{i}\xi_{y_{i}}\left(t\right)-\left(1-p_{i}\right)\xi_{a_{i}}\left(t\right)\right)\\ \frac{d\xi_{ev_{i}}}{dt}=-\frac{\partial H}{\partial ev_{i}}=k\left(\xi_{ev_{i}}\left(t\right)-\left(1-\sigma_{sym}\right)p_{i}\xi_{yv_{i}}\left(t\right)-\left(1-\left(1-\sigma_{sym}\right)p_{i}\right)\xi_{av_{i}}\left(t\right)\right)\\ \frac{d\xi_{y_{i}}}{dt}=-\frac{\partial H}{\partial y_{i}}=-C_{Y}-\nu_{i}\omega \xi_{j_{i}}\left(t\right)+\overset{8}{\underset{j=1}{\sum }}\frac{\beta_{0}u_{j}c_{ji}}{n_{i}}\left[s_{j}\left(t\right)\left(\xi_{s_{j}}\left(t\right)-\xi_{e_{j}}\left(t\right)\right)+\left(1-\sigma_{sus}\right)sv_{j}\left(t\right)\left(\xi_{sv_{j}}\left(t\right)-\xi_{ev_{j}}\left(t\right)\right)\right]+\left(\nu_{i}\omega +\left(1-v_{i}\right)\gamma_{Y}\right)\xi_{y_{i}}\left(t\right)\\ \frac{d\xi_{yv_{i}}}{dt}=-\frac{\partial H}{\partial yv_{i}}=-C_{Y}+\left\{\left(1-\sigma_{sev}\right)\nu_{i}\omega +\left(1-\left(1-\sigma_{sev}\right)v_{i}\right)\gamma_{Y}\right\}\xi_{yv_{i}}\left(t\right)-\nu_{i}\omega \left(1-\sigma_{sev}\right)\xi_{j_{i}}\left(t\right)+\overset{8}{\underset{j=1}{\sum }}\frac{\beta_{0}u_{j}c_{ji}}{n_{i}}\left[s_{j}\left(t\right)\left(\xi_{s_{j}}\left(t\right)-\xi_{e_{j}}\left(t\right)\right)+\left(1-\sigma_{sus}\right)sv_{j}\left(t\right)\left(\xi_{sv_{j}}\left(t\right)-\xi_{ev_{j}}\left(t\right)\right)\right]\\ \frac{d\xi_{a_{i}}}{dt}=-\frac{\partial H}{\partial a_{i}}=\overset{8}{\underset{j=1}{\sum }}\frac{\beta_{0}u_{j}c_{ji}b}{n_{i}}\left[s_{j}\left(t\right)\left(\xi_{s_{j}}\left(t\right)-\xi_{e_{j}}\left(t\right)\right)+\left(1-\sigma_{sus}\right)sv_{j}\left(t\right)\left(\xi_{sv_{j}}\left(t\right)-\xi_{ev_{j}}\left(t\right)\right)\right]+\gamma_{A}\xi_{a_{i}}\left(t\right)\\ \frac{d\xi_{av_{i}}}{dt}=-\frac{\partial H}{\partial av_{i}}=\overset{8}{\underset{j=1}{\sum }}\frac{\beta_{0}u_{j}c_{ji}b}{n_{i}}\left[s_{j}\left(t\right)\left(\xi_{s_{j}}\left(t\right)-\xi_{e_{j}}\left(t\right)\right)+\left(1-\sigma_{sus}\right)sv_{j}\left(t\right)\left(\xi_{sv_{j}}\left(t\right)-\xi_{ev_{j}}\left(t\right)\right)\right]+\gamma_{A}\xi_{av_{i}}\left(t\right)\\ \frac{d\xi_{j_{i}}}{dt}=-\frac{\partial H}{\partial j_{i}}=-C_{hosp}+\gamma_{hosp}\xi_{j_{i}}\left(t\right)\\ \frac{d\xi_{z}}{dt}=-\frac{\partial H}{\partial z}=0 \end{array}$$

with the transversality conditions,

$$\xi_{s_{i}}\left(T\right)=\xi_{e_{i}}\left(T\right)=\xi_{a_{i}}\left(T\right)=\xi_{y_{i}}\left(T\right)=\xi_{sv_{i}}\left(T\right)=\xi_{ev_{i}}\left(T\right)=\xi_{av_{i}}\left(T\right)=\xi_{yv_{i}}\left(T\right)=\xi_{j_{i}}\left(T\right)=0,\;\xi_{z}\left(T\right)=\theta .\left(i=1,\cdots ,\;8\right)$$

We note that θ is unknown, so an iteration process is needed to find the appropriate transversality conditions required to satisfy the isoperimetric constraints ($z\left(T\right)=B)$. This additional iterative process numerically identifies convergence problems using Newton’s method [47,48].

The Hamiltonian H is minimized with respect to the controls at the optimal rates, giving the following optimality conditions:

$${\left.\frac{\partial H}{\partial \psi_{i}}\right|}_{\psi_{i}\left(t\right)=\psi_{i}^{*}}=0.$$

By solving for $\psi_{i}$ , we obtain

$$\psi_{i}^{*}=\min\left\{max\left\{0,\;\frac{\xi_{s_{i}}-\xi_{sv_{i}}-\xi_{z}}{2C_{Vac}}\right\},\psi_{max}\;\right\}.$$
