# Supplementary material for: Vaccine Effects on Susceptibility and Symptomatology Can Change the Optimal Allocation of COVID-19 Vaccines: South Korea as an Example
Source: J Clin Med. 2021 Jun 25;10(13):2813. doi: 10.3390/jcm10132813 (PMC8268243; doi:10.3390/jcm10132813)
Supplement: Supplementary file 1 [file jcm-10-02813-s001.zip › jcm-1242360-supplementary.pdf]

Table S1. Contact matrix.

Age group	0-9	10-19	20-29	30-39	40-49	50-59	60-69	70 over
**0-9**	3.6291	0.8300	0.5731	1.4544	0.9502	0.4015	0.2046	0.0843
**10-19**	1.1550	13.2684	1.3583	1.3111	2.0143	0.7243	0.1575	0.0953
**20-29**	0.5287	1.7499	5.3694	2.6245	2.1260	1.4950	0.2523	0.0826
**30-39**	1.7398	1.3869	2.3147	5.0152	3.0261	1.5173	0.4803	0.1366
**40-49**	1.0527	2.4967	1.9639	3.0701	4.3102	1.7091	0.3557	0.1726
**50-59**	1.0196	2.2329	2.2747	2.4755	2.9717	3.0924	0.6089	0.2055
**60-69**	0.6344	0.6108	0.8605	1.5202	1.1932	1.1099	1.2349	0.2789
**70 over**	0.4525	0.7093	0.3451	0.7074	0.9602	0.7219	0.6165	0.7396

Each entry in the contact matrix (c_{ij}) corresponds to the number of age-j individuals contacted by an age-i individual per day. We used country-specific total contact matrix of South Korea from [1]. Age bins in each case were originally provided in 5-year increments, so we collapsed these to ten-year bands using population-weighted sums [2,3].

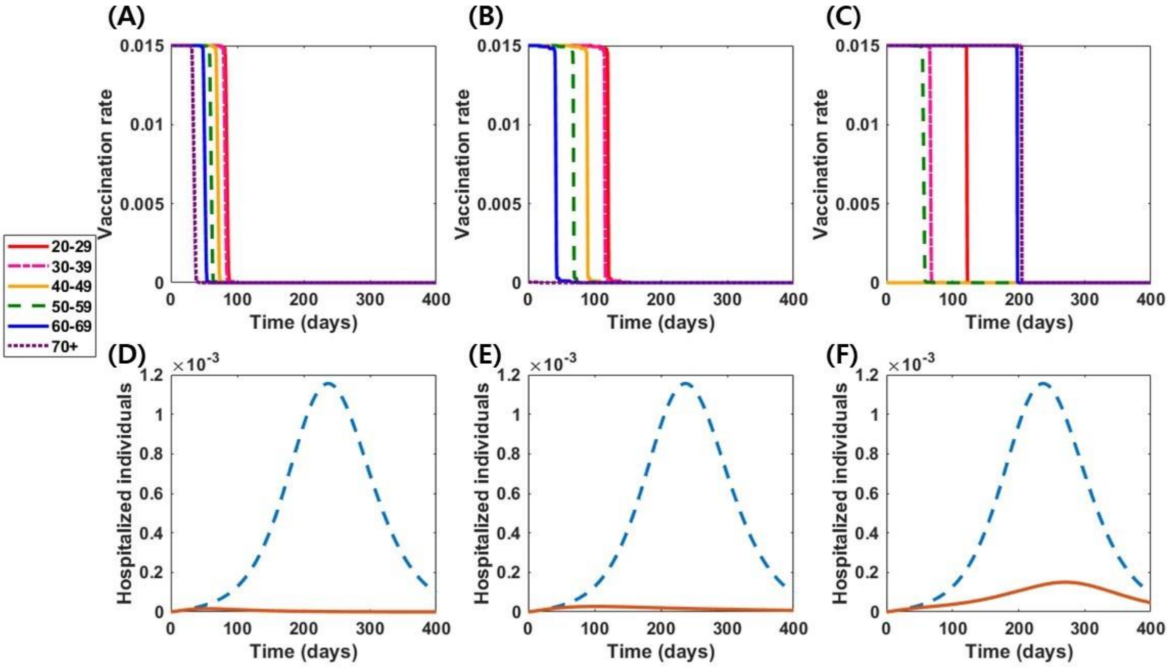

Figure S1. Optimal vaccination strategies in various vaccine scenarios. (A-C) Calculated optimal vaccination rates with vaccines 1 (A), 2 (B), and 3 (C). (D-F) Corresponding daily hospitalizations without immunization (dashed line) and with immunization (solid line) using vaccines 1 (D), 2 (E), and 3 (F).

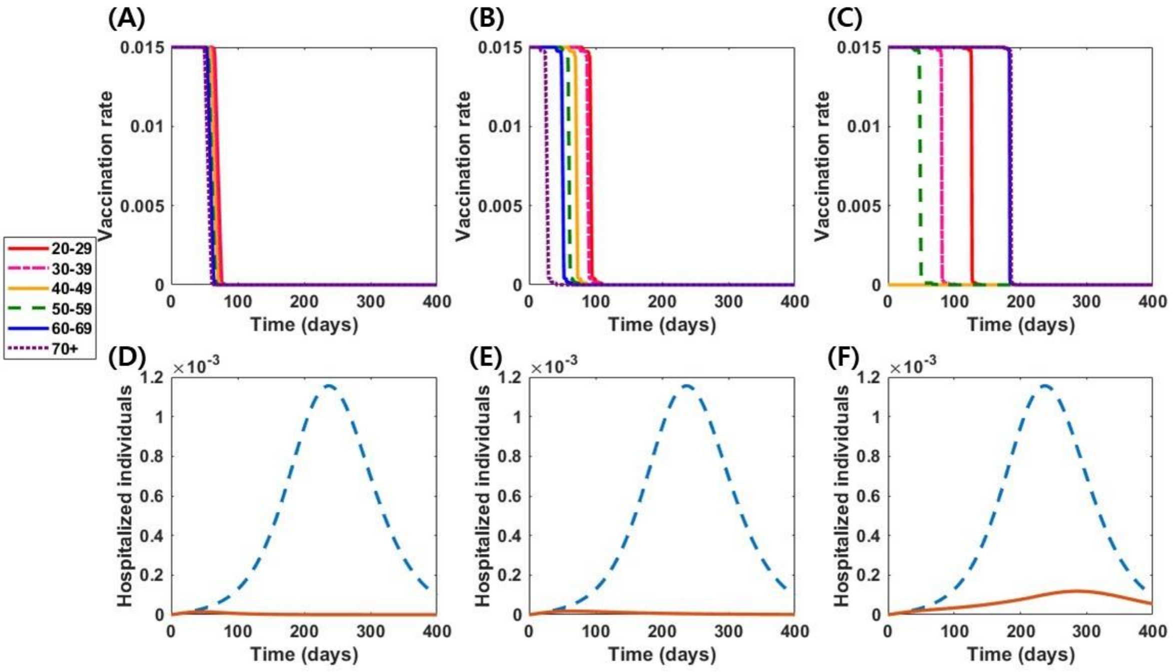

Figure S2. Optimal vaccination strategies in various vaccine scenarios. (A-C) Calculated optimal vaccination rates with vaccines 4 (A), 5 (B), and 6 (C). (D-F) Corresponding daily hospitalizations without immunization (dashed line) and with immunization (solid line) using vaccines 4 (D), 5 (E), and 6 (F).

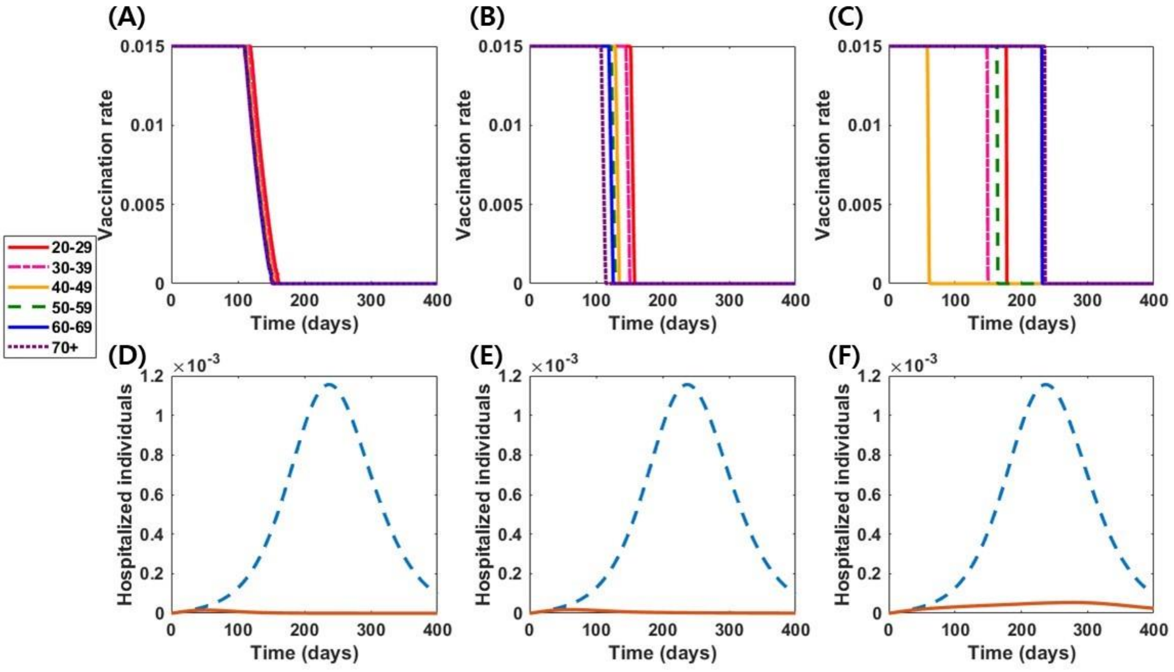

Figure S3. Optimal vaccination strategies in various vaccine scenarios with higher vaccine supply level (B=70%). (A-C) Calculated optimal vaccination rates with vaccines 1 (A), 2 (B), and 3 (C). (D-F)

Corresponding daily hospitalizations without immunization (dashed line) and with immunization (solid line) using vaccines 1 (D), 2 (E), and 3 (F).

1. Prem, K.; Cook, A.R.; Jit, M. Projecting social contact matrices in 152 countries using contact surveys and demographic data. *PLoS Comput Biol* **2017**, *13*, e1005697, doi:10.1371/journal.pcbi.1005697.
2. Bubar, K.M.; Kissler, S.M.; Lipsitch, M.; Cobey, S.; Grad, Y.; Larremore, D.B. Model-informed COVID-19 vaccine prioritization strategies by age and serostatus. *medRxiv* **2020**.
3. Buckner, J.H.; Chowell, G.; Springborn, M.R. Dynamic Prioritization of COVID-19 Vaccines When Social Distancing is Limited for Essential Workers. *medRxiv* **2020**.
